# Theta rhythm as a real-time quantitative marker for non-invasive analysis of adult neurogenesis in the intact brain

**DOI:** 10.14440/jbm.2024.0133

**Published:** 2025-05-02

**Authors:** Mahesh Kandasamy

**Affiliations:** 1 Department of Animal Science, School of Life Sciences, Bharathidasan University, Tiruchirappalli 620024, Tamil Nadu, India; 2 University Grants Commission-Faculty Recharge Programme (UGC-FRP), New Delhi 110002, India

**Keywords:** Hippocampus, Electroencephalogram, Theta rhythm, Neuroblast, Adult neurogenesis, Memory

## Abstract

**Background:**

Adult neurogenesis is a regenerative mechanism of the brain that contributes to neuroplasticity and memory consolidation. Aberrant neurogenesis is considered a key pathogenic hallmark of a wide array of neurocognitive disorders. While the functional significance of adult neurogenesis is well established in most experimental and wild animals, its occurrence in the aging human brain remains uncertain.

**Objective:**

Most studies on adult neurogenesis in humans rely on post-mortem analysis, as there is currently no method to accurately evaluate the neurogenic process in the intact brain. Theta rhythm, a neural oscillatory pattern, is believed to originate from hippocampal place cells that play a crucial role in creating cognitive maps. Theta rhythm is positively modulated by various factors, such as physical activities and enriched environment, which also promote adult neurogenesis. The strength and stability of theta rhythm are closely linked to mental well-being and cognitive functions, while its disruptions serve as indicators of neuropathogenic events that directly intersect with the regulation of adult neurogenesis.

**Conclusion:**

Modulation of the theta rhythm may reciprocally reflect the degree of neurogenesis in the adult brain, as newborn neurons can directly integrate with place cells, especially in the hippocampus. Given their electrophysical properties, newborn neurons may hold an intrinsic potential to generate theta rhythm upon motor sensory inputs and different neural activities. Biomedical tools such as electroencephalography, which measures theta rhythm, could thus be utilized to non-invasively monitor ongoing neurogenic processes in intact brains. Consequently, theta rhythm may function as a potential real-time, quantitative marker of adult neurogenesis.

## 1. Introduction

The adult brain, once believed to be a stagnant organ with limited regenerative capacity, is now recognized as a site of dynamic neurogenesis. Neurogenesis originating from neural stem cells (NSCs) has been extensively characterized in two regions of the adult brain: The hippocampus and the subventricular zone (SVZ)-rostral migratory stream (RMS)-olfactory bulb (OB) system.^1^ Evidence of neurogenesis in the cortex and hypothalamus has also been reported, and it is increasingly found to be correlated with the regulation of motor functions and endocrinological feedback mechanisms.^2^ In the SVZ, NSCs migrate along the RMS to the OB, where they differentiate into neuronal subtypes, contributing to olfactory functions.^3^ In the hippocampus, NSCs located in the subgranular zone (SGZ) have the ability to proliferate, self-renew, and differentiate into new neurons through the generation of neuroblasts.^4^ The NSC-derived neuroblasts in the SGZ, which migrate into the adjacent granule cell layer (GCL), determine the functional neurogenic process in the hippocampus.^5^ These neuroblasts mature and integrate into the hippocampal circuitry, contributing to various forms of neuroplasticity, including learning and memory.^6^ Hippocampal neurogenesis can be positively influenced by various factors such as physical exercise, enriched environments, hormones, and neuromodulators.^1^,^7^ Notably, neurogenesis in the hippocampus has been established as a potential mechanism underlying memory consolidation, with its deterioration leading to progressive memory loss and dementia.^8^,^9^ Therefore, promoting and sustaining the neurogenic process in the hippocampus of the adult brain holds great potential for restoring and boosting neuroplasticity in various emotional, psychotic, and neurodegenerative disorders.^10^,^11^ Moreover, neurogenic activities observed in the cortex, hypothalamus, and OB have been linked to sensory-motor functions, hormonal regulation, and pheromone-based behaviors, respectively.^1^

Most of the studies on the regulation of adult neurogenesis have been conducted using experimental animal models and, to a considerable extent, wild animals. Various animal experiments have provided valuable insights into the significance of neurogenesis in the modulation of neuroplasticity, its potential regulators, and neuroregenerative therapies.^4^,^12^ However, translating findings on adult neurogenesis from experimental models into the human brain remains a significant challenge. Limitations arise due to technical constraints, aging-related comorbid diseases, as well as differences in neurogenic processes between species.^13^-^15^ Given that post-mortem brain analysis is the only method for assessing neurogenesis in humans, it often fails to reflect the real-time dynamics and multifaceted stages of the neurogenic process in an active state of the brain.^13^,^16^ The lack of direct and non-invasive techniques to study neurogenesis in humans limits our understanding of its manifestations and functional significance, highlighting the need for cutting-edge methodologies or repurposing neurodiagnostic tools to bridge this gap.

The theta rhythm, a neural oscillatory pattern within the 4 – 8 Hertz (Hz) frequency range, plays a significant role in cognitive and behavioral functions, including spatial navigation-based learning and memory and emotional regulation, all of which share functional coherence with neurogenesis.^17^,^18^ The neural oscillatory pattern of theta rhythm is predominantly generated by the activity of place cells, the specialized pyramidal neurons present in the hippocampus. These place cells contribute to the establishment of cognitive maps, enabling spatial learning and memory.^19^,^20^ In the physiological state, theta rhythm is associated with motor functions, cognitive performance, and adaptive behaviors, mirroring the functional significance of adult neurogenesis.^1^,^21^,^22^ It is particularly pronounced during activities related to focused attention, exploratory behaviors, and rapid eye movement sleep.^22^-^25^ In synchrony, NSCs effectively proliferate and differentiate in response to sensory inputs, exploratory activities, and sleep, whereas a sedentary lifestyle and sleep deprivation impair neurogenesis and disrupt theta rhythm.^26^,^27^

Emerging evidence suggests that pro-neurogenic factors, such as voluntary exercise, enriched environments, music, yoga, and mental exercise, can enhance theta rhythm, facilitating and promoting neuroplasticity related to learning and memory.^1^,^28^-^31^ The strength and stability of theta rhythm are considered characteristics of mental health, while its disruptions often represent neuropathogenic events resulting from various neurocognitive diseases including Alzheimer’s disease (AD).^32^ Notably, most neurodegenerative disorders associated with dementia are characterized by impaired neurogenesis.^10^,^33^ Newly formed neurons in the hippocampus predominantly integrate into its existing neural circuits.^34^ These new neurons may directly or indirectly innervate place cells, thereby supporting the consolidation and refinement of spatial and episodic memories. Therefore, hippocampal neurogenesis can be hypothesized to directly influence the generation and stability of theta rhythms. Impaired neurogenesis in the hippocampus, observed in individuals with emotional dysregulation, psychotic disorders, and progressive memory loss, may underlie disruptions in theta rhythms. Therefore, capitalizing on the coherence between theta rhythm and adult neurogenesis could be highly conducive to the understanding of neuroregenerative plasticity and cognitive functions, and to the development of potential therapeutic interventions for neurological disorders. This review explored the reciprocal relationship between theta rhythm and adult neurogenesis, highlighting how theta activity could serve as a real-time, quantitative measure of the neurogenic process in the intact brain.

## 2. Theta rhythm: Its potential coherence with the regulation of neurogenesis in health and disease

The active neurophysiological process resulting from neuronal firing in the brain generates waves. Brain waves were first identified by German neurologist Hans Berger in the 1920s. Since then, the oscillatory patterns of neurons have been electroencephalographically (EEG) monitored to assess various activities of the brain.^35^ These brain waves signify specific oscillatory patterns of neurons linked to strategic thinking, emotion, cognition, and behavior. Five categories of wave patterns from different brain regions have been identified, ranging from low to high frequency: delta (0.5 – 4 Hz), theta (4 – 8 Hz), alpha (8 – 13 Hz), beta (13 – 32 Hz), and gamma (32 – 100 Hz).^36^ Among these, theta rhythm has been established as being intimately associated with neural activities in brain regions responsible for motor functions, learning, and memory.^37^ In 1935, Jung and Kornmüller^38^ first described a slow pattern of theta oscillation in the hippocampus of experimental rabbits. Later, in 1954, Green and Arduini^39^ demonstrated theta rhythmic harmonization between the regular activity of the cortex and the irregular activity of the hippocampus. The septal area is particularly crucial for generating theta rhythm and plays a pivotal role in transmitting signals among the OB, hippocampus, amygdala, hypothalamus, midbrain, habenula, cingulate gyrus, and thalamus.^40^ However, theta rhythm can also be generated independently within the hippocampus, without input from other brain regions.^23^,^41^ In general, hippocampal theta rhythm is associated with eye movement, respiration, and active motor behaviors, including locomotion and voluntary movements.^23^ It is a crucial component of hippocampal neuroplasticity and plays a significant role in cognition, particularly in long-term potentiation, a mechanism underlying learning and memory.^42^ Disruption of theta rhythm through pharmacological agents, electrical stimulation, or brain lesions has been shown to impair navigational efficiency in animals.^43^ For instance, in a behavioral study by McNaughton *et al.*,^44^ rats with disrupted hippocampal theta rhythms were unable to effectively learn the location of a hidden platform in the Morris water maze, a task that was principally reliant on spatial navigation skills. However, when theta-like rhythmic activity was experimentally restored in the hippocampus, their learning ability was recovered. Physical activity, such as running wheel exercise, a well-established pro-cognitive stimulus involving sensory-motor input, has been shown to increase the amplitude of theta rhythm in the hippocampus.^28^ This supports the notion that theta oscillations integrate sensory-motor input with cognitive processing, emphasizing their role in enhancing cognition and neuroplasticity, which may align with the regulation of adult neurogenesis. Brain-derived neurotrophic factor (BDNF) is well known to play a pivotal role in the molecular mechanisms underlying neurogenesis and long-term potentiation.^45^ Physical activities enhance the expression and function of BDNF in the brain, thereby improving learning and memory.^46^,^47^ Furthermore, experimental evidence suggests that the expression of BDNF in the brain may be closely associated with hippocampal theta rhythm.^48^ Moreover, theta rhythm has been shown to be modulated by electroconvulsive therapy, which induces hippocampal neurogenesis.^49^-^51^ Similarly, transcranial magnetic summation appears to enhance both neurogenesis and theta rhythm.^52^,^53^ Therefore, the potential overlap between the functional regulation of theta rhythm and neurogenesis is becoming increasingly evident.

Reduced or disrupted theta activity is associated with impaired neuronal firing, leading to the weakening of neural circuits responsible for encoding and retrieving memories. In aging and various brain diseases, abnormal theta rhythms are commonly observed, as a reflection of underlying neural dysfunction. The frequency of theta rhythm is often diminished with aging, particularly in the hippocampus, due to structural deterioration and reduced BDNF expression, thereby correlating with progressive memory decline.^54^,^55^ Theta rhythm is also reduced in affective disorders such as stress, depression, and anxiety.^56^ AD, a leading neurodegenerative condition causing dementia, is primarily driven by the abnormal accumulation of amyloid-beta plaques and tau tangles in the brain.^57^ While decreased theta rhythm is typical in AD due to hippocampal dysfunction, some studies have reported increased theta amplitude in AD subjects, suggesting an unknown compensatory mechanism.^58^,^59^ In the early progressive stages of neurodegenerative disorders, including AD and Huntington’s disease (HD), reactive neuroblastosis, a transient regenerative response to neuronal loss, has been observed and is intricately linked to neuroinflammatory mechanisms.^60^-^62^ As these diseases progress into advanced stages, the neurogenic response declines alongside worsening neuronal damage and functional deficits, ultimately leading to memory loss.^10^,^60^ Thus, reactive neurogenic activities elicited by the neuroinflammatory process in response to neuronal damage could be attributed to the proposed compensatory mechanism responsible for sensitizing theta rhythm in AD and HD. Similarly, the elevated amplitude of theta rhythm observed in epilepsy and cerebral stroke may also reflect reactive neurogenic processes, suggesting a potential common pathway influencing these electrophysiological alterations.^43^ Moreover, hallucinatory episodes during the active phases of schizophrenia have been proposed to result from reactive neuroblastosis, a process that leads to aberrant neural circuit formation and hyperactive signaling. Conversely, the failure of neuroblast integration into functional neural circuits may, in turn, contribute to cognitive impairments, such as dementia observed in the advanced stages of schizophrenia.^11^ The distinct oscillatory patterns of theta rhythm observed in individuals with schizophrenia may also be linked to varying states of neurogenic activity across different stages of the disease^63^ (Table 1).

**Table 1:** Commonalities between hippocampal theta rhythm and neurogenesis

Regulators/conditions	Target	

	Hippocampal theta rhythm	Hippocampal neurogenesis
Common positive regulators		
Voluntary exercise/physical activity	Increased	Increased
Enriched environment	Increased	Increased
Music	Increased	Increased
Meditation	Increased	Increased
Yoga	Increased	Increased
Antipsychotics	Increased	Increased
Cerebral stroke	Increased	Increased
Epileptic seizure	Increased	Increased
Electroconvulsive therapy	Increased	Increased
Transcranial magnetic stimulation	Increased	Increased
Common negative regulators		
Sleep deprivation/abnormal circadian rhythm	Disrupted	Impaired
Aging	Disrupted	Declined
Anxiety	Disrupted	Impaired
Depression	Disrupted	Impaired
Stress	Disrupted	Impaired
Schizophrenia	Disrupted	Disrupted
Alzheimer’s disease	Disrupted	Impaired
Parkinson’s disease	Disrupted	Impaired
Huntington’s disease	Disrupted	Impaired
Bipolar disorder	Disrupted	Impaired
Pathogenic alcohol consumption	Disrupted	Impaired

## 3. Theta rhythm as a functional measure of ongoing neurogenesis in the intact brain: Potential implications for human studies

The hippocampus is organized into distinct layers containing densely packed neuronal populations and their processes, including the dentate gyrus (DG), molecular layer, cornu ammonis (CA) 3, CA2, CA1, hilus, and subiculum. It receives input from the entorhinal cortex, which projects to the DG through the perforant fiber pathway and to CA3 through the mossy fiber pathway.^64^ CA3 neurons further project to CA1 through the Schaffer collateral pathway.^65^ Place cells, a subset of pyramidal neurons located primarily in the CA1 and CA3 regions, become active when an animal explores specific locations, thereby contributing to spatial navigation.^66^ Pacemaker neurons, specialized subtypes in the medial septum, intrinsically generate rhythmic bursts through voltage- and time-dependent ion fluxes.^67^ These pacemaker neurons synchronize with hippocampal place cells, driving the neural oscillations that underlie theta rhythm.^23^ Notably, hippocampal theta rhythm can also be generated, independent of external feedback from other brain regions, suggesting the presence of intrinsic oscillatory mechanisms within the hippocampus itself.^23^,^68^

Table 1 highlights the regulation of hippocampal theta rhythm and neurogenesis by both intrinsic and extrinsic factors, as well as in health and disease.

Neuroblasts are considered as immature neurons with electrophysiological properties that originate from NSCs in the SGZ and integrate into the GCL of the DG.^61^ These neuroblasts extend axons to the CA3 region and dendrites into the molecular layer.^1^,^69^ CA3 pyramidal neurons, in turn, project to the DG, hilus, and CA1 through Schaffer collaterals.^65^,^70^ The circuit dynamics between CA1 pyramidal neurons and DG granule cells involve both feedforward and feedback mechanisms mediated by excitatory and inhibitory synaptic inputs.^71^ As neuroblasts mature and integrate into hippocampal circuits, their electrical and synaptic activities may contribute to the generation or modulation of theta rhythm, and they can innervate place cells. An increased number of neuroblasts can positively influence the activity of place cells, while reduced neurogenesis may impair this function. Therefore, the stability and amplitude of theta oscillations could serve as real-time markers of the neurogenic process, reflecting the maturation and integration of new neurons into the hippocampal network. These oscillatory changes may provide insights into the role of neurogenesis in cognitive functions, such as learning and memory. Understanding the potential overlap between theta rhythm and neurogenesis may thus enhance our comprehension of the mechanisms underlying brain plasticity in both health and disease and support the development of novel diagnostic measures.

**Figure 1:**
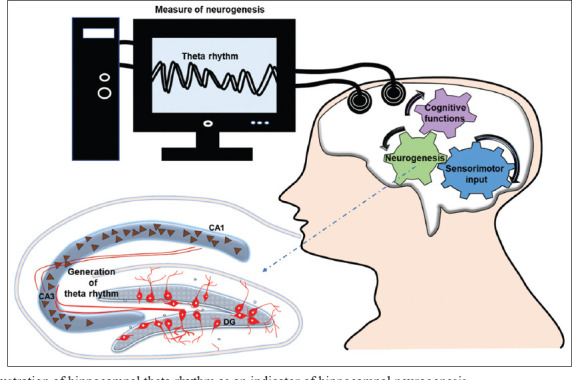
Schematic illustration of hippocampal theta rhythm as an indicator of hippocampal neurogenesis Abbreviations: CA: Cornu ammonis; DG: Dentate gyrus.

The incidence of neurogenesis has been documented in neurogenic niches within the brains of various animal models, including insects, amphibians, birds, rodents, and non-human primates. In 1998, Erickson and colleagues from Fred Gage’s group provided groundbreaking evidence of neurogenesis in the adult human brain using bromodeoxyuridine labeling.^55^ This method demonstrated the generation of new neurons in the adult human hippocampus. Despite this scientific milestone, subsequent studies have reported conflicting results regarding the existence and functional relevance of adult neurogenesis in humans. These discrepancies are partly attributed to methodological limitations, such as potential misidentification of cell types and practical challenges in post-mortem analysis.^13^ Efforts to assess neurogenesis in humans have included imaging techniques, such as magnetic resonance imaging (MRI) and positron emission tomography scans, which provide functional and structural insights but lack direct evidence of neurogenic activity.^72^ To date, no non-invasive method has been established that directly mirrors the status of neurogenic activities in the intact human brain. Among various techniques, EEG, a non-invasive tool for measuring brain activity, holds a promising strategy in linking theta rhythms to the functionality and integration of newly generated neurons in the adult brain.^73^ Combining EEG with advanced methods, such as magnetoencephalography, functional MRI, diffusion tensor imaging, and neurochemical assays may offer a multidimensional perspective on the relationship between neurogenesis and theta rhythmicity.^74^ This integrative approach could elucidate neuroplasticity mechanisms underpinning cognition in both health and disease, while also informing novel therapeutic interventions aimed at restoring hippocampal function by enhancing neurogenesis and modulating theta rhythms. Such approaches may hold promise for treating conditions, such as Parkinson’s disease, AD, HD, epilepsy, and mood disorders. However, implementing EEG to correlate theta rhythm with neurogenesis requires foundational studies to establish its validity. At present, no direct evidence has confirmed a relationship between EEG-based readouts of theta rhythm and the neurogenic process. Rahsepar *et al*.^75^ demonstrated that the activation of engram neurons leads to phase-specific stimulation relative to theta oscillations in the CA1 region. Previously, we postulated that neuroblasts generated in the adult brain play a crucial role in mediating and facilitating the function of engram, a hypothetical biophysical parameter of memory consolidation and processing.^61^ Considering that engram neurons in the hippocampus are reportedly generated through neurogenesis, a recent study by Lei *et al*.^76^ further demonstrated that adult neurogenesis plays a key role in recruiting new engrams responsible for encoding new memories, rather than relying solely on pre-existing engrams. Lacefield *et al*.^77^ showed that experimentally abolishing the neurogenic process through focal irradiation altered different oscillatory patterns, including hippocampal theta rhythm in the brain. Moreover, whole-cell patch-clamp recordings conducted by Pernía-Andrade and Jonas^78^ found that granule neurons, typically generated through *in vivo* neurogenesis in the hippocampal DG, predominantly exhibited theta rhythm over gamma coherence. In addition, Rendeiro and Rhodes^79^ provided seminal evidence that physical activity-mediated enhancement of theta rhythm is strongly associated with the generation of new neurons. Taken together, these findings support the idea that the generation of theta rhythm is a distinct phenomenon during circuit formation in the hippocampus, closely tied to the integration of newly-born neurons. Furthermore, several lines of experimental evidence suggest that neuroblasts exhibit electrophysiological properties in the brain that are detectable through EEG. Electrophysiological techniques such as patch-clamp recordings have confirmed that newly-generated neuroblasts in the adult brain display spontaneous and evoked electrical activity.^80^,^81^ Based on these observations, neuroblasts may contribute to theta oscillations through two potential mechanisms: (i) by activating place cells upon integration into the CA regions of the hippocampus or (ii) by generating theta oscillations independently due to their intrinsic electrophysiological properties. Therefore, theta rhythm could serve as a physical parameter of engrams, potentially generated by newly-born neurons, regardless of place cell activation (Figure 1).

Experimental manipulation of theta rhythm appears to differentially impact the generation of newly-born neurons in the brain. A genetically modified mouse model expressing a mutant phospholipase C (PLC) gene were characterized by the absence of a subset of theta rhythms and exhibited various behavioral deficits, including sleep abnormalities.^82^ Interestingly, PLC mutant animals also showed enhanced levels of neurogenic activity in the hippocampus, leading to a schizophrenia-related phenotype,^83^ which aligns with our recent report suggesting that the reactive neurogenic process might be an underlying mechanism of schizophrenia.^11^ This enhanced neurogenesis could represent a compensatory response to an impaired hippocampal network resulting from disrupted theta rhythm. While feedback mechanisms are typically generated through the hippocampal-septal loop to maintain neurogenesis in balance, the elimination of theta rhythm may disrupt this loop, resulting in a withdrawal of feedback regulation on neurogenesis. Thus, results from the PLC mutant mouse model provide evidence that the loss of theta rhythm may lead to disorganized hippocampal circuits, lifting inhibitory feedback on the neurogenic process and triggering reactive neurogenesis. This phenomenon could be attributed not only to schizophrenia-like behaviors but also to early phases of neurodegenerative disorders, which are often characterized by enhanced theta oscillations and reactive neuroblastosis. Although the occurrence of theta is predominantly reported in the hippocampus, theta-like oscillations have also been observed in other regions such as the lateral hypothalamus, ventromedial hypothalamus, suprachiasmatic nucleus, and the OB.^84^,^85^ Theta rhythm in hypothalamic regions has been linked to sleep cycles, hormonal feedback mechanisms, and thermoregulation.^84^ Olfactory theta rhythm, primarily driven by respiration and pheromones is functionally connected to hippocampal activity, influencing memory and spatial navigation.^86^ While theta rhythm has been reported to originate from various sub-neuronal populations, independent of place cells, both the hypothalamus and OB have also been identified as neurogenic regions. Therefore, it can be hypothesized that the generation of new neurons in the hypothalamus and OB may also contribute to the generation of theta rhythms.^3^,^26^,^87^ This suggests that adult neurogenesis in these regions could modulate region-specific forms of neuroplasticity through theta oscillations. However, direct evidence linking adult neurogenesis in the hypothalamus and OB to the theta rhythm generation remains scarce. Although both processes have been extensively studied, they have largely been investigated separately, leaving a gap in understanding how newly-generated neurons in these regions might contribute to or be influenced by theta activity. Future research integrating electrophysiological, molecular, and imaging techniques is needed to establish a mechanistic link between neurogenesis and theta rhythm. Indeed, the hypothesis proposed in this review presents a novel perspective, suggesting that newborn neurons in various regions of the adult brain may contribute to the modulation and shaping of brain oscillations.

## 4. Conclusion

The endogenous activation of NSCs and the subsequent integration and survival of neuroblasts in the adult brain have emerged as promising restorative therapeutic approaches. However, the assessment of real-time neurogenesis in the human brain remains a significant challenge. The theta rhythm, a brainwave oscillation within the 4 – 8 Hz frequency range, is extensively studied in the hippocampus. Theta activity is closely associated with hippocampal-dependent learning and synaptic plasticity – processes that significantly overlap with adult neurogenesis. Both neurogenesis and theta rhythm are regulated by similar intrinsic and extrinsic factors in health and disease, emphasizing their interdependence. The integration of newly- formed neuroblasts from the GCL into functional circuits involving place cells in the CA3 and CA1 subfields of the hippocampus is likely to underlie the generation and modulation of theta rhythms. Non-invasive techniques, such as EEG could potentially be implemented to monitor hippocampal theta activity and correlate it with ongoing neurogenesis in the intact brain, paving the way for application in human studies. In addition, theta rhythms have also been observed in the hypothalamus and OB, which are recognized as additional sites of adult neurogenesis. Considering the electrophysiological potential of newborn neurons, it is plausible that theta rhythms may be directly generated by neurogenic activities. Thus, this article emphasizes that neurogenesis may provide the basis for theta rhythm generation, either by activating place cells or by directly contributing to theta oscillatory activity upon integration into existing neural circuits within neurogenic regions. Nevertheless, this hypothesis requires further validation to establish its feasibility, accuracy, and translational potential in both pre-clinical and clinical settings. Future studies utilizing optogenetic, chemogenetic, or Cre-LoxP-based selective elimination of place cells in the CA regions of the hippocampus could be highly instrumental in disrupting theta rhythms. This approach would allow researchers to investigate whether neurogenesis-derived neurons independently contribute to theta oscillations, as observed through EEG, providing further insights into the relationship between neurogenesis and theta rhythm. Furthermore, EEG-based experiments using hippocampal slice cultures treated with pro-neurogenic and anti-neurogenic factors could provide further validation. Ultimately, MRI combined with electrophysiological correlates of hippocampal function related to neurogenesis, or using pharmacological regimens that simultaneously modulate theta rhythm and neurogenic process in the intact human brain during various conditions, could further elucidate these mechanisms. Finally, recent advancements in three-dimensional brain organoids developed from induced pluripotent stem cell-derived neurons, when integrated into EEG-based experiment models, may offer crucial validation for the proposed role of neurogenesis in theta rhythm generation.

## Data Availability

Not applicable.
